# Tumour Cell Seeding to Lymph Nodes from In Situ Colorectal Cancer

**DOI:** 10.3390/cancers15030842

**Published:** 2023-01-30

**Authors:** Maria Teresa Rodrigo-Calvo, Karmele Saez de Gordoa, Sandra Lopez-Prades, Ivan Archilla, Alba Diaz, Mario Berrios, Jordi Camps, Eva Musulen, Miriam Cuatrecasas

**Affiliations:** 1 Pathology Department, Centre of Biomedical Diagnosis (CDB), Hospital Clinic, 08036 Barcelona, Spain; 2 Molecular Pathology of Inflammatory Conditions and Solid Tumours Research Group, Institut d’Investigacions Biomèdiques August Pi i Sunyer (IDIBAPS), 08036 Barcelona, Spain; 3 Centro de Investigación Biomédica en Red en Enfermedades Hepáticas y Digestivas (CIBEREHD), 28029 Madrid, Spain; 4 Department of Basic Clinical Practice, University of Barcelona (UB), 08036 Barcelona, Spain; 5 Pathology Department, Hospital Universitario Central de Asturias, 33011 Oviedo, Spain; 6 Gastroenterology Department, Hospital Clinic, University of Barcelona, 08036 Barcelona, Spain; 7 Department of Cell Biology, Physiology and Immunology, Faculty of Medicine, University Autonomous of Barcelona, 08193 Bellaterra, Spain; 8 Pathology Department, Hospital Universitari General de Catalunya-Grupo QuironSalud, Sant Cugat del Vallès, 08195 Barcelona, Spain; 9 Josep Carreras Leukaemia Research Institute, Badalona, 08916 Barcelona, Spain

**Keywords:** in situ colorectal cancer, lymph node, staging, diagnosis, OSNA

## Abstract

**Simple Summary:**

Lymph node (LN) metastasis is an important prognostic factor in colorectal cancer (CRC). We aimed to search for lymphatic vessels (LVs) in the lamina propria of 39 surgically resected in situ CRC, as well as to detect the presence of tumour burden in the regional LNs. We identified the presence of LVs in the mucosa of all tumours (39/39; 100%) using D240 immunostains. LNs were analysed by both H&E and a RT-PCR-based molecular method. All cases were pN0 with H&E, and the molecular assay detected the presence of low amounts of tumour burden in the LNs of 11/39 (28%) cases, with no clinical consequences at 1 to 5 years of follow-up. The amount of tumour burden in LNs has proven to be a prognostic factor. Despite the fact that pTis is considered to have little or no risk of LN metastasis, our results enabled to quantify the amount of tumour burden within LNs, which may help clinical management.

**Abstract:**

Lymph node (LN) metastasis is an important prognostic factor in colorectal cancer (CRC). We aimed to demonstrate the presence of lymphatic vessels (LV) in the mucosa of in-situ (pTis) CRC, and of detectable tumour burden in regional LNs. This is an observational retrospective study of 39 surgically resected in situ CRCs. The number of LVs was evaluated in both pTis and normal mucosa using D2-40 immunostains. All LNs were assessed with both H&E and the One Step Nucleic Acid Amplification (OSNA) assay, and the results were correlated with clinicopathological features. D2-40 immunohistochemisty revealed LVs in the lamina propria of all pTis CRC (100%), being absent in normal mucosa. A median of 16 LNs were freshly dissected per patient, and all cases were pN0 with H&E. Molecular LN analysis with OSNA revealed the presence of low amounts of tumour burden in 11/39 (28%) cases (range 400 to 4270 CK19 mRNA copies/µL), which had no clinical consequences. This study demonstrates the presence of LVs in the lamina propria in 100% of pTis CRC, as well as the presence of low amounts of tumour burden in regional LNs, only detected by molecular methods. Given the prognostic value of LN tumour burden, its molecular quantification may help a patient’s clinical management.

## 1. Introduction

It has long been established that in situ colorectal carcinoma (CRC) has scant capacity for lymph node (LN) metastasis, which is supported by the hypothesis that the normal colonic mucosa is devoid of lymphatic vessels (LV) [1,2]. In addition, one of the most important prognostic factors in CRC is the presence of LN metastasis. Nevertheless, in early stages of CRC, the presence of LN micrometastases may be difficult to detect by standard hematoxylin and eosin (H&E) analysis, which has caused researchers to seek for more sensitive methods of LN analysis [3].

The colonic mucosa has abundant capillary vessels in the lamina propria, ensuring a rich blood supply. Its lymphatic drainage is indirectly observed by the large number of LV in the submucosa. Nevertheless, LVs are not seen in normal colorectal mucosa. Fogt et al. used D2-40 immunohistochemistry (IHC) to seek for the presence of LV in the normal colonic mucosa, adenomas, and invasive carcinomas. They found that LVs were absent in normal colon mucosa, but present in the lamina propria of in situ and invasive carcinomas [1]. The same observations were validated by other authors [2,4]. However, other studies have shown that there are pathological conditions, including neoplasms and inflammation, in which an induction of lymphatic angiogenesis occurs [5], with presence of LV in the lamina propria of the colorectal mucosa. All these studies demonstrate that LV may be present in the lamina propria of the colon in abnormal conditions other than frankly invasive carcinomas [1,2,5,6].

The One Step Nucleic Acid Amplification (OSNA) is an RT-PCR-based molecular assay for cytokeratin 19 (CK19) mRNA detection. It is a quantitative and highly sensitive method, useful for the diagnosis of LNs metastases and quantification of the amount of tumour burden in the LN compartment. This method of LN assessment has been validated for the analysis of sentinel LNs from breast carcinoma, as well as for LN staging in CRC [7,8]. In the later, it has been proven to be more accurate than conventional H&E for LN staging, and to have prognostic implications in early stages of CRC [9,10,11]. 

The purpose of our study was to demonstrate the presence of LVs in the colorectal mucosa of in situ CRC, and to determine both the capability of tumour cell seeding into regional LNs, and the ability to detect and quantify the amount of tumour burden within regional LNs.

## 2. Materials and Methods

### 2.1. Patients and Tumours

This is a retrospective and observational study from a single institution for molecular identification of LN metastases. From May 2012 to October 2018, we included 312 stage I to III CRC patients submitted to surgery after a diagnosis of carcinoma or unresectable adenoma, in the context of population-based CRC screening program using the fecal immunochemical occult blood test (FIT) (OC-Sensor^®^, Eiken, Japan). Among all patients included, we selected all pTis CRC. LN analysis from the resected specimens was performed with both H&E and OSNA. 

Recruited cases were from patients with primary surgery for an endoscopically unresectable adenoma which resulted in a pTis CRC. All pTis tumours were entirely submitted for histological examination and step sections were performed to ensure the absence of submucosal infiltration. All tumours invaded only the lamina propria, and none invaded either partially or totally the muscularis mucosae. Thus, although our tumours were in situ carcinomas according to the AJCC and the College of American Pathologists definition of pTis, i.e., “tumours involving the lamina propria and/or muscularis mucosae, but not extending through it (intramucosal carcinoma)” [12,13], none of our cases reached the muscularis mucosae. Inclusion criteria were patients older than 18 years-old with tumour positivity for CK 19 IHC. Exclusion criteria were CRC with pT stage higher than pTis, metastatic carcinomas, presence of other neoplasms, familial adenomatous polyposis, synchronous tumours, or chronic inflammatory bowel disease. 

### 2.2. Ethical Considerations

The study was approved by the Ethics and Scientific Committee of our institution (Ref. HCB 2012/7324). All patients signed and kept a copy of the informed consent document for participation in the study after the nature of the research was fully explained. Another copy was kept with the patient’s clinical files, and a third copy was kept in the Biobank files of our institution.

### 2.3. Immunohistochemistry Staining 

Immunohistochemical staining with CK19 (A53-B/A2, ref 760-4281, Roche, Basel, Switzerland), CAM5.2 (ref 790-4555, Roche, Basel, Switzerland), and podoplanin (D2-40) (D2-40, ref 760-4395, Roche, Basel, Switzerland) antibodies was performed with the Ventana Benchmark Ultra (Roche, Basel, Switzerland) on 2-micron tissue sections placed on FLEX IHC microscope slides, according to the manufacturer’s protocol, with 20 min incubation for each primary antibody [14]. Immunostains for CK19 and D2-40 were performed in both normal mucosa and pTis from all cases. Positivity for CK19 was defined as membranous staining of more than 10% of tumour cells. D2-40 membrane or cytoplasm positivity was located on the lymphatic endothelial cells. CAM5.2 was performed in all pTis to evaluate the intratumour tumour budding, and for an enhanced identification of tumour cells within the LVs.

### 2.4. Lymphatic Vessel Assessment

We assessed the presence and density of LVs in both tumours and normal mucosa on the D2-40-stained slides. All pTis slides were first scanned at low power with an optical microscope (Olympus BX41, Olympus, Japan) to find the area of the tumour with the highest density of LVs. Then, an LV count was performed using the 20× objective lens, corresponding to an area of 0.785 mm^2^ [5,15,16]. Only LVs located in the lamina propria were counted, excluding LV in contact with the superficial part of the muscularis mucosae, which are known to be present in normal colon mucosa.

### 2.5. Intratumour Budding Evaluation

We counted the number of intratumour tumour buds (ITB) present in the lamina propria of pTis with cytokeratin immunostains, using the classification of the International Tumour Budding Consensus Conference (ITBCC), i.e., low TB (Bd1, from 0 to 4 buds), intermediate TB (Bd2, 5 to 9 buds), and high TB (Bd3, ≥10 buds) [17]. We furthermore included the recently described Bd0 category when there was a complete absence of tumour buds, redefining Bd1 as 1 to 4 buds [18]. 

### 2.6. Lymph Node Dissection and Analysis

LNs from pericolic and intermediate levels (D1 and D2) of pTis CRC were freshly dissected within 45 min after surgical resection, and were not separated into stations. Fresh dissection was performed as described by Rakislova et al. [19]. Briefly, the mesocolon or mesorectum was separated from the colorectal wall using sterile scalpel and forceps. Fresh LNs were isolated and cut in half. One half of the LN was put into a PCR tube for OSNA analysis using the pooling protocol [19]. The other half was used for conventional pN stage with H&E and processed with formalin fixation and paraffin embedding (FFPE).

### 2.7. One Step Nucleic Acid Amplification (OSNA) Assay

The OSNA assay is a PCR-based quantitative amplification of CK19 mRNA. It uses the RT-LAMP (Real-Time Loop Mediated Isothermal Amplification) at 65 °C without previous purification steps. It is a fast and standardized method used for routine sentinel LN analysis in breast carcinoma, which has also been validated for CRC LN staging [7,8,9]. The results are given as the total tumour load (TTL), defined as the total amount of CK19 copies/µL present within all the LNs from a given surgical specimen [20]. The TTL represents the amount of tumour present in all the LNs analyzed from one surgical specimen. An OSNA result of ≥250 copies/µL was considered positive. Confirmation that the primary tumour was positive for CK19 immunohistochemistry was necessary to guarantee a negative OSNA result. 

### 2.8. Lymph Node Staging

In all patients, pathological LN diagnosis (pN stage) was performed using conventional H&E analysis, according to the American College of Pathology/TNM-UICC protocols [13] (https://documents.cap.org/protocols/ColoRectal.Bx_4.2.0.1.REL_CAPCP.pdf, accessed on 26 January 2023). Both pathologists and surgeons were blinded to the molecular OSNA results, which did not interfere with the patient’s clinical management.

### 2.9. Statistical Analysis 

The χ^2^ test was used to analyze the association between qualitative variables, followed by Fisher’s exact test and Student’s *t*-test, or by the Mann-Whitney test for quantitative variables. A *p* < 0.05 was considered significant.

## 3. Results

### 3.1. Study Sample 

Thirty-nine in situ CRC surgical specimens were included in the study from May 2012 to October 2018. None of the cases had had a previous endoscopic resection. The mean age of the patients was 68.6 years-old (range 56–89) and 59% were males. Tumours ranged in size from 0.4 to 5.5 cm. Tumours ≥ 2 cm were arbitrarily defined as big. All tumours were low grade and most (31/39; 79%) were on the right colon. The CK19 immunostain resulted in positives in all pTis CRC. Patients did not receive adjuvant therapy and were alive without disease between 1 and 5 years of follow-up. Clinical and pathological characteristics of all cases are described in Table 1. 

### 3.2. The Normal Colonic Mucosa Is Devoid of Lymphatic Vessels

**Figure 1:**
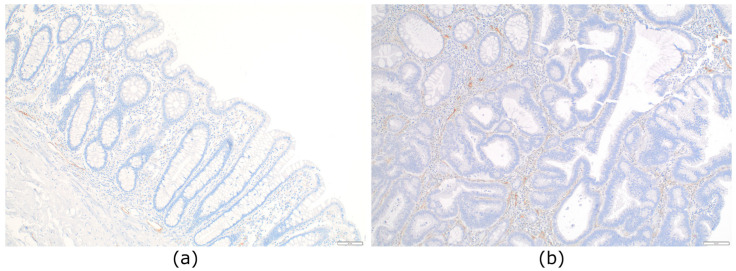
Distribution of lymphatic vessels in normal colon mucosa and in situ colorectal carcinoma: (**a**) Normal colonic mucosa devoid of lymphatic vessels in the lamina propria. Lymphatic vessels are seen just above the muscularis mucosae and in the submucosa (D2-40, ×100); (**b**) lymphatic vessels present in the lamina propria of a pTis colorectal carcinoma (D2-40, ×100).

D2-40 immunochemistry performed on normal colorectal mucosa demonstrated the absence of LVs in the lamina propria in all cases, but presence next to the muscle fibers of the superficial part of the muscularis mucosae, without reaching the base of the crypts. Abundant LVs were present in the submucosa (Figure 1a). 

### 3.3. Presence of Lymphatic Vessels in the Mucosa of In Situ CRC

When assessing the presence of LVs of in situ CRC using D2-40 IHC, we observed LVs in the lamina propria of all tumours (39/39; 100%). We next quantified the number of LVs present in each tumour, which ranged from 1 to 7. We arbitrarily defined two groups: low-LV density when containing 0–4 LVs and high-LV density when containing 5–7 LVs (Figure 1b). We only counted the intratumoural LVs. The density of LVs was not significantly different depending on location, gender, or size (Table 2). We did not identify any tumour cell inside LVs with CK stains (CAM5.2 or CK19).

### 3.4. Intratumoural Tumour Budding in pTis

**Figure 2:**
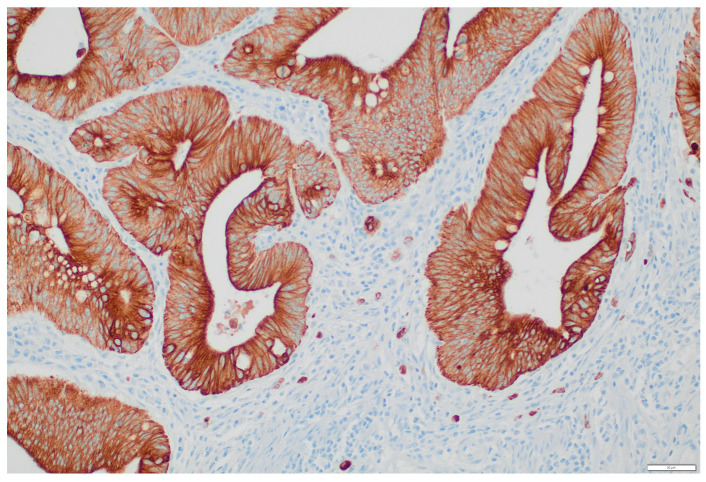
Tumour budding of an in situ colorectal carcinoma showing intermediate budding (Bd2) (CK CAM 5.2 ×200).

Intratumoural tumour budding (ITB) was assessed with CK CAM 5.2. We divided ITB into two groups, ITB-negative (Bd0) and ITB-positive (Bd1 and Bd2). Most cases, 76.9% (30/39) had no tumour buds and were classified as Bd0. Nine cases (23.1%) were listed as ITB-positive (Figure 2), seven had Bd1, and two had Bd2. We did not find any differences between the OSNA results and tumour budding, nor with the number of LVs (*p* = 0.217 and *p* = 0.923, respectively).

### 3.5. Detection of Tumour Burden in Regional LNs with the OSNA Assay

A total of 648 LNs were dissected from 39 cases, with a mean of 16.6 LNs per patient. 556 LNs were freshly dissected and 92 (14.1%) were found post formalin fixation. Fresh LNs were analyzed by both H&E and the OSNA assay. All patients were pN0 by H&E. OSNA detected the presence of tumour burden in the LNs of 11/39 (28%) cases, with low TTLs (range 400 to 4270 CK19 mRNA copies/µL). There was no significant correlation of OSNA positive cases with age, sex, location, and LV density (Table 3).

### 3.6. Patient’s Follow-Up

Regarding clinical outcome, all patients were alive without disease, with a median follow-up of 49.62 months (range 13–86 months).

## 4. Discussion

This is a novel molecular study demonstrating the presence of tumour burden in regional LNs at very early stages of CRC. In all cases LNs were negative with H&E analysis (pN0), and the presence of low amounts of tumour burden within LNs was detected only by molecular methods. We also observed the presence of LVs in the lamina propria in all in situ CRC. 

In situ CRC are non-invasive carcinomas, considered to have limited risk of LN metastasis [12]. Nevertheless, using LN molecular analysis, we have demonstrated the presence of small amounts of tumour load within the LNs of 28% pTis CRC. The presence of LVs in the colonic mucosa in inflammatory and neoplastic conditions has been previously demonstrated through a mechanism of lymphangiogenesis [5]. Accordingly, some studies unexpectedly identified metastatic spread in more than 1% of so-called “in situ” colorectal adenocarcinomas and have reported the capacity of pTis CRCs to metastasise to LNs. There are also single reports of lymphatic invasion in “intramucosal CRC”. In their opinion, it is biologically and clinically plausible that a small percentage of in situ CRC acquire the ability of LN dissemination without the necessity of submucosal invasion, since the lymphatic plexus is known to be present just above and within the muscularis mucosa. Thus, they claim that “intramucosal” CRCs with disruption or invasion through the muscularis mucosa can invade the LVs [21,22]. Lewin et al. reported 15 “intramucosal” poorly differentiated colorectal carcinomas originated in endoscopically resected adenomas, of which one case developed metastatic disease, and another was associated with Paget’s disease of the anal skin. The other 13 cases were alive without disease with a median follow-up of 13 months [23]. Shia et al. described two cases. One was an intramucosal poorly differentiated adenocarcinoma with 8 negative lymph nodes, which recurred 17 months after surgical resection, involving the omentum and the liver. The second case was an intramucosal adenocarcinoma originated on a tubulovillous adenoma, with the foci of lymphatic invasion at the base of the mucosa but not beyond the muscularis mucosae [24].

To our best knowledge, this is the first study including a series of surgical specimens of in situ CRCs in which LN assessment has been performed with both H&E and molecular methods. Further, LNs from normal colons and rectums are normally small-sized. Similarly, LN metastases from early CRC are usually small and located in LNs of ≤5 mm, which are difficult to identify. In this regard, Aldecoa et al. demonstrated that those LNs which are more prone to harbour tumour burden are those from the lymphatic basin which drain the tumour, usually located close to the tumour [25]. Another interesting finding of our work is that all in situ CRC had at least one LV in the lamina propria, demonstrated with D2-40 immunostaining. All our cases were confined to the mucosa, without invasion of the muscularis mucosae, did not have high-grade features, and were all pN0 by H&E. We found presence of tumour burden in the LNs of 11/39 cases (28%) by molecular analysis. This finding indicates that in situ CRCs are capable of invading intramucosal LVs more often than expected, being able to seed some tumour cells into regional LNs. Although we could not see any lymphovascular invasion in none of the tumours with CK immunostains, nine tumours had ITB, seven of them Bd1 and two Bd2, which we assume it is the way tumour cells reached the LVs. Additionally, most of our cases 76.9% (30/39) had Bd0, that is, complete absence of tumour budding. In this regard, Zlobec et al. showed that pT1 tumours with Bd0 are less aggressive than tumours with Bd1 [18]. In our cohort, the subgroup of in situ CRC with Bd0 had neither lesser number of LVs nor lower amounts of TTL in their LNs. As far as we know, there are no studies of pTis CRC and ITB. We neither found any significant correlation between the number of LVs present in the mucosa, presence of ITB, or any other clinicopathological factors, nor with OSNA positivity. 

In our series, the presence of a low quantity of tumour load in regional LNs of in situ CRC had no clinical significance. In this regard, it is important to emphasize that it has been demonstrated that the TTL correlates with the pN, and that the amount of tumour burden in the LNs, or TTL, has prognostic value. Yamamoto demonstrated that the TTL increases with the pN stage of CRC, having 1550 copies/µL for pN0 cases, 24,050 copies/µL for pN1, and 90,600 copies/µL for pN2 cases [9]. In a recent study from our group, Archilla et al. validated these results, with similar values, finding 1775 copies/µL for pN0, 49,413 copies/µL for pN1 and 95,000 copies/µL for pN2 CRC. In fact, the TNM rules are based on the number of positive LNs, defined as tumour metastases ≥ 0.2 mm in size. Regarding the high sensitivity of molecular LN testing, the low TTL values probably correspond to ITCs rather than genuine metastases. These results are presumably reliable, and, if reported, would correspond to pN0 being the LN tumour burden detected only by molecular means. Our results confirm that even in pN0 CRC, a small amount of tumour burden is present within the LNs but most likely with no clinical consequences. A plausible explanation for the latter is that the mesocolic or mesorectal fat, which contains regional LNs, is removed with the surgical procedure. Thus, a regional lymphadenectomy is performed in curative-intended surgery, and with it, ITC and small groups of tumour cells not detected with H&E are removed, which may have no clinical consequences. Several studies have demonstrated a greater sensitivity in the identification of small amounts of tumour burden, i.e., isolated tumour cells or micrometastases with the use of the OSNA assay [9,20,26]. Molecular LN testing using quantitative methods is very useful since it gives the information of the amount of tumour burden within the LNs, which can guide the patient’s clinical management. In addition, the TTL has been found to be a prognostic factor in CRC, with values of ≥6000 copies/µL associated with worse overall survival (OS) and disease-free survival (DFS) [10,11]. Since none of our cases had neither LN metastases nor disease recurrence, our results could reinforce the current position that in situ carcinoma should be managed as for high grade dysplasia rather than invasive carcinoma. In addition, surgical removal of these lesions is associated with the morbidity and mortality of major surgery, which ranges from 3 to 20% [27]. In fact, colorectal cancer screening programmes have led to an increase in the detection of early CRC, including pTis, pT1 and pT2 stages. There are few studies regarding the recurrence rate of pTis, which are limited to rectal carcinomas, being 2.6% at five years and 6.7% at 10 years, and a cumulative incidence of systemic recurrence of 0.8% at 5 years [28]. Even if the findings of our study may be of scientific relevance, they clearly demonstrate that tumour seeding into the LNs occurs at a very early stage of CRC development and should be taken into consideration.

One limitation of our study is that we have used half of the LN for the OSNA assay and the other half for H&E analysis, which may have induced a tumour allocation bias. Despite that, some tumour burden was identified in the part of the LN used for molecular analysis. Another limitation is that there was no statistical association between budding and vessel density/higher tumour burden by OSNA, or other clinicopathological features. Given the number of cases in our cohort, the analysis is probably underpowered.

## 5. Conclusions

In agreement with previous reports, we have also confirmed the presence of LVs in the lamina propria of pTis CRC. In addition, we have demonstrated that tumour cells from in situ CRC can seed the LNs, probably in the form of isolated tumour cells or small groups of cells, which confer no additional risk for these patients. The relevance of this study is that molecular assays enable to quantify the amount of tumour burden present in the LN compartment, which has been demonstrated to have prognostic value. In the setting of pTis CRC, the TTL can help patient’s clinical management.

## Figures and Tables

**Table 1:** Clinical and pathological characteristics of in situ colorectal carcinomas.

Category	No (Total)	No (%)
Gender		
Male	23	59
Female	16	41
Age (year)		
<65	17	44
≥65	22	56
Tumour size (cm)		
<2	23	59
≥2	16	41
Tumour location		
Right colon	31	79
Left colon and rectum	8	21
Lymphatic vessels		
Low LV	25	64
High LV	14	36
Tumour budding		
TB-negative (Bd0)	30	77
TB-positive (Bd1, Bd2)	9	23
OSNA		
Positive	11	28
Negative	28	72
LN		
Fresh	556	86
Formol	92	14

LV (lymphatic vessels), TB (Tumour budding), OSNA (One Step Nucleic Acid Amplification), LN (lymph node).

**Table 2:** Clinicopathologic characteristics of pTis colorectal carcinomas according to lymphatic vessel density.

Category	High-LV (Number)	Low-LV (Number)	*p*
Gender			
Male	2	21	0.538
Female	3	13	
Age (year)			
<65	1	16	0.428
≥65	3	19	
Tumour size (cm)			
<2	3	20	0.491
≥2	1	15	
Tumour location			
Right colon	3	28	0.176
Left colon and rectum	2	6	
Tumour budding			
TB-negative (Bd0)	3	27	0.923
TB-positive (Bd1, Bd2)	1	8	

LV (lymphatic vessels), TB (Tumour Budding).

**Table 3:** Characteristics of pTis colorectal carcinomas according to OSNA results.

Category	OSNA + (Number)	OSNA – (Number)	*p*
Gender			
Male	7	16	0.711
Female	4	12	
Age (years)			
Mean	65.72	69.78	
<65	6	11	0.387
≥65	17	5	
Tumour size (cm)			
Mean	2.8	1.9	
<2	5	18	0.293
≥2	6	10	
Tumour location			
Right colon	9	26	0.307
Left colon and rectum	2	2	
Lymphatic vessels			
Mean	2.8	2.3	
Low LV	9	25	0.357
High LV	2	3	
Tumour budding			
TB-negative (Bd0)	7	23	0.217
TB-positive (Bd1, Bd2)	4	5	

OSNA (One Step Nucleic Acid Amplification), LV (lymphatic vessels), TB (Tumour budding).

## Data Availability

The datasets generated and/or analyzed during the current study are available from the corresponding author on reasonable request.
